# A Definition

**Published:** 1869-11

**Authors:** 


A Definition.—The compositor at this office finds *Traumatic* defined in Johnson's and Worcester's Dictionaries "A medicine to heal wounds—useful for wounds." Dunglison, as we know, gives a different meaning. The true interpretation it may prove desirable for medical disputants round about to settle.

